# The relationship between dietary inflammatory index and all-cause and cardiovascular disease-related mortality in adults with metabolic syndrome: a cohort study of NHANES

**DOI:** 10.3389/fendo.2024.1417840

**Published:** 2025-01-10

**Authors:** Qunwei Ma, Ying Zhang, Daowen Zhang, Cancan Liu, Weiwei Zhu, Guixia Wang, Nannan Xu, Xue Zhang, Rui Huang, Huijun Zhang, Shuhang Xu, Chao Liu, Kuanlu Fan

**Affiliations:** ^1^ Department of Endocrinology, The Second Affiliated Hospital of Xuzhou Medical University, Xuzhou, Jiangsu, China; ^2^ Department of Endocrinology, The Affiliated Hospital of Integrated Traditional Chinese and Western Medicine, Nanjing University of Chinese Medicine, Nanjing, China

**Keywords:** all-cause mortality, cardiovascular disease-related mortality, metabolic syndrome, dietary inflammation index, National Health and Nutrition Examination Survey

## Abstract

**Objective:**

This study aims to investigate the correlation between dietary inflammatory index (DII) and mortality resulting from all-cause and cardiovascular diseases (CVD) in adults affected by metabolic syndrome (MetS).

**Methods:**

The focus of this study was to analyze the information of 13,751 adults who had been diagnosed with MetS. DII scores were computed based on a 24-hour dietary intake at the start of the study. By implementing both the Cox regression analysis and restricted cubic spline(RCS) analysis, we examined the correlation between DII score and mortality.

**Results:**

After a mean follow-up duration of 114 months, a total of 2,343 individuals (representing 13.45% of the sample) died, with 639 fatalities attributed to CVD. The degrees of dietary inflammation were classified into three groups based on DII scores: low, medium, and high-grade. The mortality rates for each tertile of DII were 11.55%, 13.96%, and 15.05%, respectively. In comparison to participants with T1, the multivariate-adjusted hazard ratios (HR) and 95% confidence intervals (CI) for participants with T3 were 1.16 (95% CI: 1.01-1.34) regarding mortality caused by any reason, and 1.26 (95% CI: 0.95-1.68) for mortality related to CVD. Through the use of the Kaplan-Meier survival curve and RCS, it was observed that individuals in the high DII tertile had an increased likelihood of death compared to those in the low DII tertile.

**Conclusion:**

Our findings provide validation of the theory that diets high in inflammatory substances contribute to elevated mortality rates for all causes and CVD-related deaths in individuals diagnosed with MetS.

## Introduction

Metabolic syndrome(MetS), a prevalent metabolic disorder caused by the rising rates of obesity (1), has garnered increasing attention in recent years. In the United States, the occurrence of MetS is reported to be as high as 35% in adults and 50% in individuals aged 60 and above (2). The risk of health outcomes has been identified in MetS and is strongly associated with increased mortality rates for all causes (3), particularly cardiovascular disease (CVD) mortality, which is the primary factor contributing to death in individuals with MetS (4). Consequently, implementing robust and efficient strategies to avert the onset of MetS, slow its advancement, and enhance survival rates is essential.

MetS is a disorder caused by chronic inflammation in the body, which has been observed to initiate it. This ongoing inflammation is strongly associated with the occurrence of diabetes (DM) and hypertension (HT) (5, 6). Compelling evidence indicates that dietary choices can significantly influence the body’s inflammatory state. Unhealthy eating habits promote an excessive release of pro-inflammatory cytokines while simultaneously diminishing the synthesis of anti-inflammatory agents. This imbalance causes the immune response to remain persistently activated, leading to an increased inflammatory burden on the body (7, 8). The impact of certain dietary elements, like unsaturated fats, fiber, and vitamins, has been demonstrated in reducing body inflammation and lowering the chances of developing MetS. Therefore, evaluating the risk factors for MetS requires consideration of the inflammatory effects associated with people’s dietary patterns.

Consequently, the dietary inflammatory index (DII) was developed as a quantitative approach to assess dietary inflammation levels, emerging as the situation demanded. This index can illustrate the overall diet of a person on a scale ranging from the most anti-inflammatory to the most pro-inflammatory (9, 10). A study following a group of 3521 adults with a normal body mass index(BMI) demonstrated that a diet scoring high on the DII significantly heightened the risk of CVD mortality (11). Furthermore, findings derived from the NHANES database uncovered that dietary patterns marked by diminished DII scores were linked to a reduced probability of death from any origins among individuals aged 60 years and older (12).

Investigating the relationship between DII and mortality among patients with MetS is crucial; however, there is a deficiency of comprehensive studies on this topic. In order to address this research gap, the present study utilizes data gathered from the NHANES to assess the association between the DII and mortality in individuals diagnosed with MetS. Our analysis has the potential to provide fresh insights into diverse realms, encompassing dietary supervision, nutritional epidemiology, and MetS treatment.

## Methods

### Study participants

**Figure 1:**
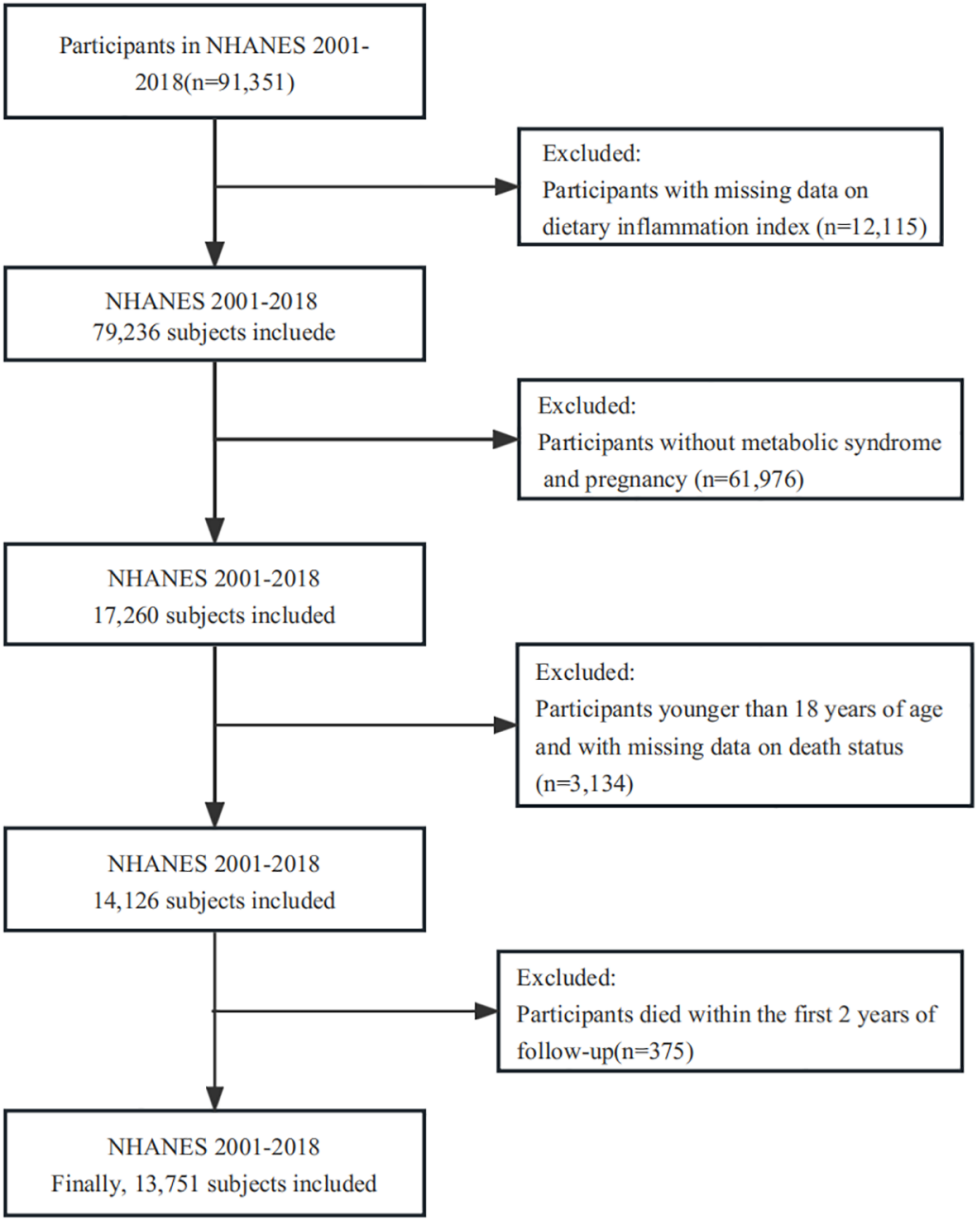
Flow chart of study participants.

The NHANES database is an ongoing survey in the United States that focuses on nutrition and health among the population. The survey employed advanced sampling techniques to guarantee the representativeness of the data, instead of using a basic random sample from the general population. To conduct this study, we examined data from 9 survey cycles spanning from 2001 to 2018, which encompassed a total of 91,351 individuals. However, 12,115 participants had to be excluded from the analysis because their data was incomplete for calculating DII. Additionally, we eliminated participants (n = 61,976) whose data did not meet the criteria for defining MetS (refer to Ascertainment of MetS). We further excluded individuals under the age of 18 and those with missing survival data (n = 3134). In order to mitigate the issue of reverse causality, we additionally eliminated individuals who passed away within a span of 2 years during the follow-up period (n = 375). In totality, our investigation consisted of a comprehensive group of 13,751 participants (see **Figure 1**).

National Centre for Health Statistics Institutional Ethics Review Board approved the study, and a written consent was taken from all the participants. The data used in the present study were downloaded from NHANES website (https://www.cdc.gov/nchs/nhanes/index.htm).

### Ascertainment of MetS

MetS is defined according to the NCEP ATP III-2005 criteria, which specify that an individual must have three or more of the following factors: increased waist circumference, heightened blood pressure, decreased high-density lipoprotein cholesterol (HDL-C), amplified triglycerides (TG), and increased fasting glucose (13). Specifically, increased waist circumference is defined as equal to or exceeding 102 cm in males and 88 cm in females. Heightened blood pressure is indicated by a blood pressure equal to or surpassing 130/85 mm Hg or the use of medication for hypertension. Decreased HDL-C is characterized by levels below 40 mg/dL in males and below 50 mg/dL in females, or the use of specific treatment for reduced HDL-C. Amplified TG levels are defined as equal to or surpassing 150 mg/dL, or the use of medication for elevated TG. Increased fasting glucose is indicated by levels of 100 mg/L or higher, or the use of medication for elevated glucose and a previous diagnosis of type 2 diabetes.

### Calculation of the DII

The DII score system, which was formulated by Shivappa based on an extensive analysis of existing literature, serves as a valuable tool for assessing the potential inflammatory impact of dietary components (10). In our study, we calculated the DII by considering a total of 27 specific nutrients, encompassing carbohydrates, cholesterol, protein, fiber, polyunsaturated fatty acids, folic acid, saturated fat, n-3 fatty acids, n-6 fatty acids, overall fat content, alcohol, monounsaturated fatty acids, as well as vitamins A/B12/C/D/E, thiamin, caffeine, magnesium, niacin, riboflavin, zinc, iron, selenium, beta-carotene, and energy composition. Previous investigations have shown that the predictability of DII calculations remains unaffected when utilizing less than 30 food parameters (14). For a thorough understanding of how the DII is calculated, please refer to the **Supplementary Method** section.

### Outcome variables

The determination of mortality status was accomplished by employing a distinctive research identifier and conducting probabilistic matching with the National Death Index until December 31, 2019. Causes of death were categorized based on the ICD-10 codes. The main measure of interest was all-cause mortality, encompassing deaths from any cause within the duration of the study. Moreover, we regarded CVD-related mortality as a secondary outcome, encompassing fatalities caused by cerebrovascular disorders (I60–I69) and different heart-related ailments (I00–I09, I11, I13, and I20–51).

### Covariates

The demographic information collected for this study encompassed age, sex, race, education attainment, smoking and alcohol status, as well as family poverty-income ratio(PIR). To assess the participants’ health, physical examinations and laboratory tests were conducted, which included measurements of BMI, waist circumference, systolic blood pressure(SBP) and diastolic blood pressure(DBP), levels of HDL-C, serum creatinine (Scr), glycated hemoglobin (HbA1c), albumin (Alb), gamma-glutamyl transpeptidase (GGT), alanine aminotransferase (ALT), aspartate aminotransferase (AST), and uric acid (UA). Additionally, the presence of comorbidities such as hyperlipidemia (HL), DM, HT, and CVD were identified. The specific definitions for these factors can be found in **Supplementary Table S1**.

The missing data in the study were addressed using a multilevel approach designed for survey data (15, 16). The Jomo package was utilized to generate five imputed data sets. A burn-in of 1000 iterations and 1000 updates were conducted to guarantee stochastic independence between the imputed data sets.

### Statistical analysis

We followed the analytical guidelines provided by the Center for Disease Control and Prevention and accounted for the complex multistage cluster survey design of the NHANES by applying appropriate sample weights to each participant during statistical analyses. We presented categorical variables by proportions, and for continuous variables, we reported them as mean (standard error). To evaluate differences between groups, a weighted chi-square test was employed for categorical variables, while we performed an analysis of variance for continuous variables.

The DII was utilized to evaluate its impact on mortality risk through the application of multivariate Cox proportional hazards models, treating the DII as both a continuous variable and a tertile parameter. Mortality was designated as the dependent variable, while the DII functioned as the independent variable, with the first tertile group serving as the control. *A priori* potential confounders for adjustment in the multivariable regression models were selected based on established knowledge of clinically relevant factors and their associations with dietary exposures and outcomes (17, 18). Model 1 did not incorporate any corrections, whereas Model 2 made adjustments for factors including sex, educational level, and smoking habits. Building upon Model 2, the third model implemented further corrections for additional confounding variables, such as PIR, BMI, CVD, and alcohol consumption. To analyze the data, linear trends were assessed by treating the median of each category as a continuous variable. Furthermore, a dose-response curve was created employing a restricted cubic spline (RCS) function to investigate the association between DII and mortality risk in patients with MetS. The Kaplan-Meier method was utilized to estimate the survival rates among different groups, and a log-rank test was conducted to make comparisons.

To assess the robustness of our results, we conducted stratified analyses considering age (≥60 years or <60), sex (male or female), race (non-Hispanic white or other), education attainment (less than high school, high school or equivalent, and college or above), BMI (<25, 25-30, or ≥30 kg/m2), and PIR (<1, 1-2.9, or ≥3). To examine the significance of the interaction effect between the stratification variables and the DII, we conducted a wald test. All statistical analyses in this investigation were conducted using the R software, version 4.1.2. Statistical significance was determined by a two-sided p-value of less than 0.05.

## Results

### Baseline characteristics of study participants

This research involved a total of 13,751 individuals from the NHANES, representing a noninstitutionalized population of approximately 63.58 million residents in the United States. We classified DII scores into low (T1: −5.28–1.06), moderate (T2: 1.07–2.76), and high inflammation levels (T3: 2.77–5.79), with T1 as the reference group (**Table 1**). To provide a summary of the basic characteristics related to DII and all-cause mortality, please refer to **Table 1**, **Supplementary Table S2**, respectively. Females represented approximately 52.95% of the weighted percentage in this research, and the mean age was 53.74 ± 0.22 (weighted mean) years. During the 114-month follow-up period, a significant number of participants, totaling 2,343, were identified as having died from various causes. The occurrence of all-cause mortality was found to be 15.05% within the group adhering to a high inflammation diet, a significantly greater value than the 11.55% observed in the low-inflammatory diet group. The data provided in **Supplementary Table S2** further supports these findings, demonstrating significantly higher DII scores (P < 0.0001) among patients who died during the follow-up period.

**Table 1:** Baseline characteristics of participants from NHANES 2001–2018.

Characteristic	Overall (N=13,751)	Dietary inflammatory index			*P* value
		T1(N=4610)	T2(N=4573)	T3(N=4568)	
Age, years	53.74(0.22)	53.82(0.34)	53.53(0.32)	53.87(0.38)	0.66
Sex, n (%)					< 0.0001
Female	7449(52.95)	1967(40.37)	2484(54.60)	2998(65.49)	
Male	6302(47.05)	2618(59.63)	2097(45.40)	1587(34.51)	
Educational attainment					< 0.0001
Under high school	4086 (19.20)	1122(14.65)	1357(19.56)	1607(24.06)	
High school or equivalent	7399(59.36)	2433(56.41)	2526(60.74)	2440(61.28)	
College or higher	2266(21.44)	1055(28.94)	690(19.70)	521(14.66)	
Race, n (%)					0.14
Non-Hispanic White	6435(70.72)	2190(71.42)	2139(71.47)	2106(69.13)	
Others	7316(29.28)	2395(28.58)	2442(28.53)	2479(30.87)	
Family poverty-income ratio					< 0.0001
PIR<1	6242(39.72)	1957(35.92)	2062(39.53)	2223(44.24)	
PIR 1~2.9	2901(14.37)	767(11.12)	935(13.21)	1199(19.30)	
PIR≥3	4608(45.92)	1861(52.97)	1584(47.26)	1163(36.46)	
BMI, kg/m^2^					0.002
BMI<25	4124(28.53)	1400(29.76)	1427(29.96)	1297(25.60)	
BMI 25~30	896(5.89)	285(5.19)	271(5.62)	340(6.97)	
BMI≥30	8731(65.58)	2900(65.04)	2883(64.42)	2948(67.43)	
Smoking status, n (%)					< 0.0001
Never	4109(30.34)	1496(33.82)	1393(30.70)	1220(25.99)	
Former	6990(50.00)	2416(51.89)	2294(49.44)	2280(48.45)	
Now	2652(19.66)	673(14.29)	894(19.86)	1085(25.56)	
Alcohol status					< 0.0001
Never	3116(19.87)	854(17.15)	1040(19.29)	1222(23.58)	
Former	2274(17.68)	826(18.46)	780(18.17)	668(16.26)	
Mild	4340(35.16)	1672(39.95)	1416(33.98)	1252(30.96)	
Moderate	1707(13.87)	567(13.30)	598(14.81)	542(13.50)	
Heavy	2314(13.43)	666(11.14)	747(13.75)	901(15.69)	
Diabetes diagnosis, n (%)					0.33
DM	5453(32.57)	1747(31.76)	1802(31.78)	1904(34.34)	
IFG	1123(8.59)	382(8.58)	365(8.68)	376(8.51)	
IGT	492(3.63)	172(3.95)	144(3.16)	176(3.76)	
No	6683(55.21)	2284(55.71)	2270(56.39)	2129(53.39)	
Hypertension diagnosis, n (%)	9278(64.55)	3080(65.03)	3078(64.42)	3120(64.14)	0.83
CVD diagnosis, n (%)	2607(16.20)	730(14.27)	870(15.97)	1007(18.65)	< 0.001
Hyperlipidemia diagnosis, n(%)	12907(94.52)	4268(94.22)	4312(94.15)	4327(95.27)	0.18
Waist circumference (cm)	110.69(0.20)	111.12(0.27)	110.70(0.34)	110.18(0.35)	0.09
SBP (mmHg)	128.32(0.24)	128.27(0.43)	128.30(0.36)	128.40(0.41)	0.96
DBP (mmHg)	73.11(0.22)	74.13(0.28)	72.96(0.33)	72.10(0.34)	< 0.0001
HbA1c(%)	6.05(0.02)	6.02(0.02)	6.04(0.02)	6.09(0.03)	0.08
Albumin(g/dl)	4.20(0.01)	4.25(0.01)	4.19(0.01)	4.15(0.01)	< 0.0001
Alanine aminotransferase(U/L)	28.66(0.31)	30.55(0.41)	28.79(0.75)	26.37(0.36)	< 0.0001
Aspertate aminotransferase(U/L)	26.11(0.18)	27.04(0.29)	25.96(0.30)	25.23(0.27)	< 0.0001
Serum creatinine(umol/l)	81.40(0.38)	81.34(0.50)	81.60(0.57)	81.26(0.78)	0.91
Gamma-glutamyltransferase (U/L)	34.68(0.49)	35.20(0.78)	34.63(0.86)	34.14(0.90)	0.68
Uric acid (umol/l)	351.18(1.10)	354.98(1.78)	349.90(2.02)	348.22(1.60)	0.01
HDL-C(mmol/l)	1.13(0.00)	1.12(0.01)	1.14(0.01)	1.15(0.01)	0.01
Death, n(%)	2343(13.45)	699(11.55)	784(13.96)	860(15.05)	<0.01

BMI, body mass index; SBP, systolic blood pressure; PIR, poverty-to-income ratio; DBP, diastolic blood pressure; IFG, impaired fasting glycaemia; IGT, impaired glucose tolerance; DM, diabetes; CVD, cardiovascular diseases; HbA1c, glycated hemoglobin; HDL-C, high-density lipoprotein cholesterol; T, tertiles.

The prevalence rates for HT, DM, HL, and CVD were 64.55%, 32.57%, 16.2%, and 94.52%, respectively. Individuals with T3 appeared to face a greater risk of CVD (18.65% vs. 14.27%, p < 0.0001) in comparison to those with T1. People who followed a proinflammatory diet showed a tendency toward being female, having an education level below high school, and exhibiting higher HDL-C levels alongside lower RIP, DBP, Alb, AST, ALT, and UA levels. Furthermore, these participants exhibited a greater propensity for being present-day tobacco consumers, overdrinker, or overweight (all p < 0.05).

### Association between DII and all-cause mortality

**Figure 2:**
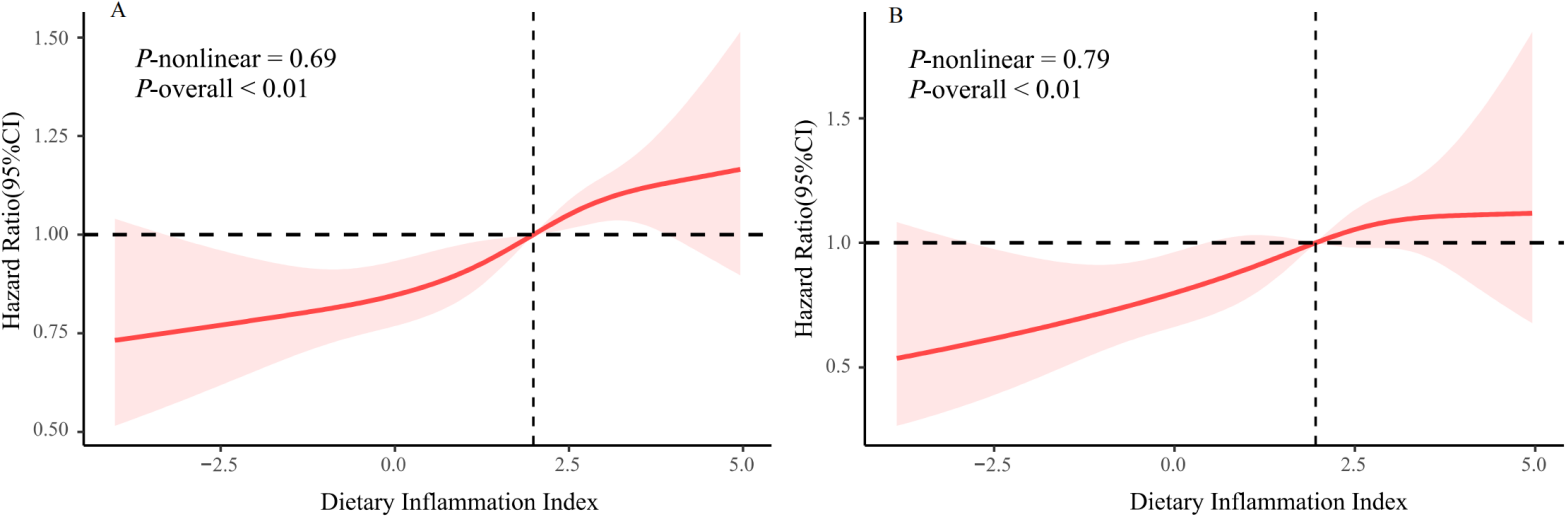
The restricted cubic spline plot of the association between DII with **(A)** all-cause mortality **(B)** CVD-related mortality.

In this cohort, we observed an association between the DII score and all-cause death risk (**Table 2**). The analyses on continuous DII score revealed a 7% (95% CI: 1.03–1.11) higher hazard of death with each 1-unit growth of the DII score, without other confounders adjusted. The death risk persisted when confounders were fully adjusted (HR: 1.04, 95% CI: 1.01–1.08). When examining DII by tertiles, participants with the highest DII score were prone to gain greater risk of death as compared with those with the lowest tertile (HR: 1.30, 95% CI: 1.12–1.51), without other confounders adjusted. The association persisted after potential confounders were further controlled (HR: 1.16, 95% CI: 1.01–1.34). Furthermore, assessments utilizing DII tertile as a continuous variable indicated a significant p value for the trend in the adjusted model (*P* for trend = 0.04). Additionally, the RCS plot curve illustrated a linear positive correlation between DII and all-cause mortality, suggesting that higher DII scores significantly increased the risk of all-cause mortality (P for nonlinearity = 0.69; see **Figure 2A**).

**Table 2:** HR (95% CI) for all-cause mortality according to dietary inflammatory index in patients with MetS from NHANES 2001-2018.

Characteristic	Model 1		Model 2		Model 3	
	HR(95%CI)	*P*	HR(95%CI)	*P*	HR(95%CI)	*P*
All-cause mortality						
DII(continuous)	1.07(1.03,1.11)	<0.001	1.06(1.03,1.10)	<0.001	1.04(1.01,1.08)	0.01
DII tertiles						
T1	ref		ref		ref	
T2	1.14(1.00,1.30)	0.04	1.11(0.97,1.27)	0.12	1.11(0.96,1.27)	0.15
T3	1.30(1.12,1.51)	<0.001	1.26(1.09,1.47)	0.002	1.16(1.01,1.34)	0.04
*P* for trend		<0.001		0.002		0.04
Cardiovascular mortality						
DII(continuous)	1.10(1.03,1.17)	0.005	1.10(1.03,1.18)	0.01	1.07(1.01,1.13)	0.02
DII tertiles						
T1	ref		ref		ref	
T2	1.05(0.79,1.39)	0.74	1.05(0.78,1.41)	0.75	1.06(0.79,1.41)	0.71
T3	1.41(1.05,1.87)	0.02	1.43(1.06,1.92)	0.02	1.26(0.95,1.68)	0.11
*P* for trend		0.02		0.02		0.11

DII, dietary inflammatory index; T, tertiles; HR: hazard ratio; 95% CI: 95% confidence interval.

^2^Model 1: Nonadjusted.

^3^Model 2 was adjusted for sex, educational attainment, smoking status.

^4^Model 3 was further adjusted for PIR, BMI, CVD and alcohol drinking.

### Association between DII and CVD-related mortality

The results for CVD-related mortality (**Table 2**) showed similar findings. Our observations indicated that individuals who are inclined to consume a pro-inflammatory diet exhibited a markedly elevated risk of mortality related to CVD compared to the risk of mortality from all causes. The analysis of the continuous DII score showed a 7% (95% CI: 1.01-1.13) increase in the hazard of CVD-related death for each 1-unit increase in the DII score, following full adjustments for confounding variables. When the DII was analyzed in tertiles, those participants with the highest DII scores were found to have a greater likelihood of experiencing CVD-related mortality (HR: 1.41, 95% CI: 1.05–1.87). Nevertheless, after comprehensive adjustments for confounding factors, this disparity was not statistically significant. Additionally, as illustrated in **Figure 2B**, we observed a linear relationship between DII and CVD-related mortality (P for nonlinearity = 0.79), suggesting that elevated DII scores are associated with an increased risk of death from CVD.

### Survival analysis

**Figure 3:**
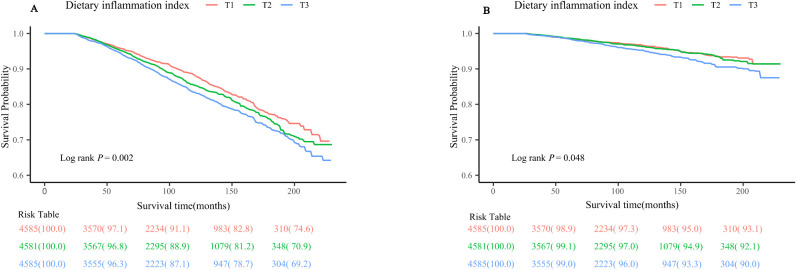
Kaplan-Meier survival curve for **(A)** all-cause mortality **(B)** CVD-related mortality. CVD, cardiovascular disease.

**Figure 3** illustrates the Kaplan-Meier survival curves for individuals categorized into DII tertiles. It is evident from the graph that the group classified as T3 is associated with the highest risk of mortality caused by all-causes. Additionally, this group also demonstrates an increased risk of mortality related to cardiovascular disease. The statistical analyses further support these findings, with Log-rank P values of 0.002 and 0.048 for all-causes mortality and cardiovascular disease-related mortality, respectively.

### Subgroup analysis

**Figure 4:**
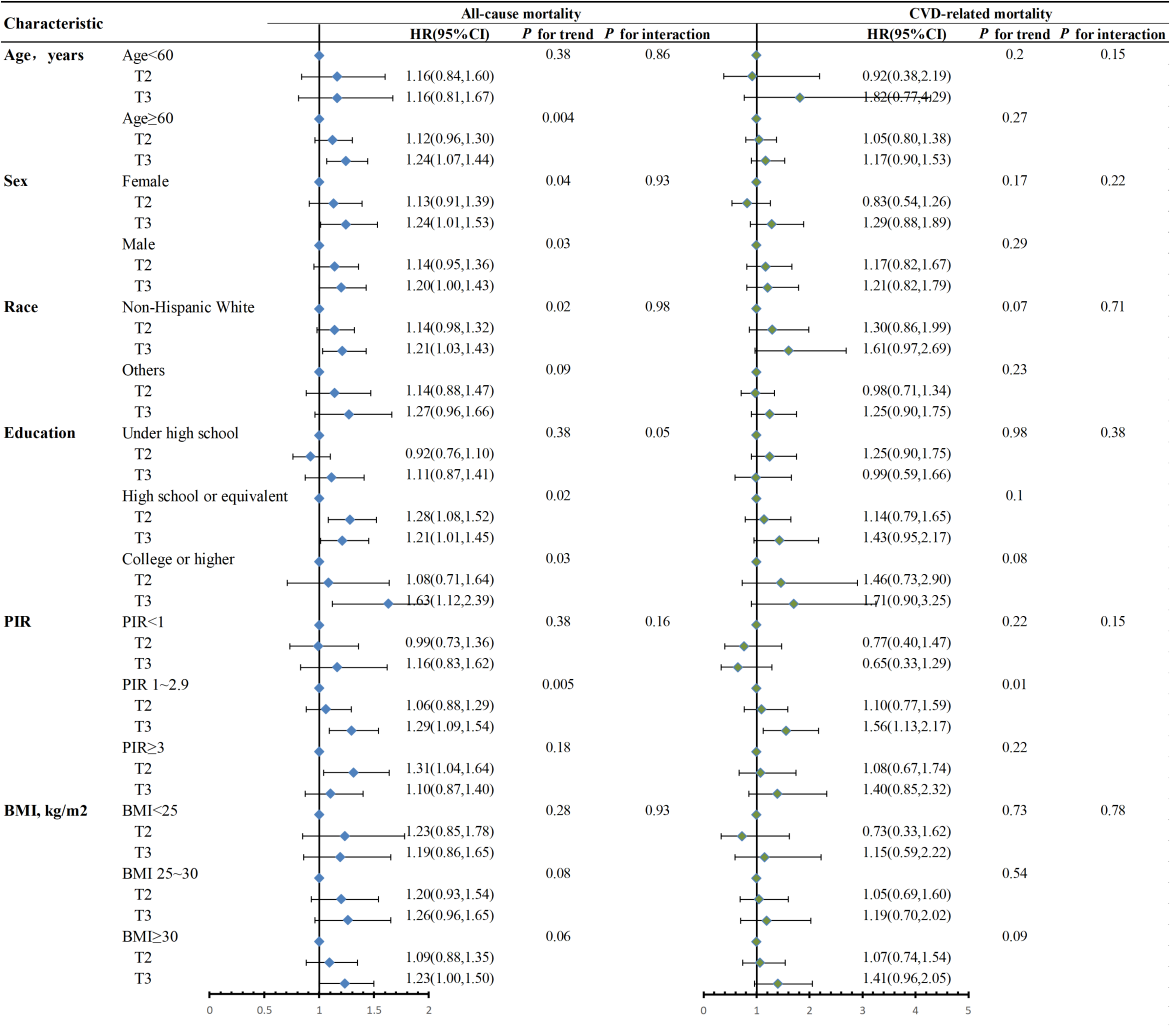
Stratified analysis of the association between DII and mortality in MetS patients in NHANES 2001-2018. Adjusted for age, sex, race, education, BMI, PIR. When stratified, stratum variables are not included in the model. The Wald test was performed to examine the interaction between DII and stratification variables. BMI, body mass index; PIR, poverty-to-income ratio; T, tertiles; HR, hazard ratio; 95% CI, 95% confidence interval.

The variables of age, sex, race, education, PIR and BMI were stratified into groups, and the examination validated that DII had positive correlations with mortality from any cause in the majority of subgroups (**Figure 4**). Additionally, we further explored the association of DII with CVD-related mortality stratified by these variables (**Figure 4**), and the results displayed a similar trend. No significant interactions were observed between DII and these variables (P for all interactions > 0.05).

## Discussion

Our research established a connection between the DII scores of individuals with MetS and both overall mortality and mortality related to CVD. Pro-inflammatory dietary patterns emerged as a significant risk factor for the mortality of these patients. Individuals adhering to a dietary pattern associated with increased inflammation, as indicated by elevated DII scores, experienced an elevated risk of mortality from all causes as well as from CVD. In contrast to individuals in group T1, individuals in group T3 exhibited a 30% increased chance of mortality from any cause and a 41% elevated probability of mortality related to CVD. Even after accounting for possible confounding variables, elevated DII scores continued to show a significant relationship with overall mortality. RCS analysis, survival analysis, and stratified analysis further demonstrated the robustness of these findings.

Chronic systemic inflammation has been demonstrated to be linked with MetS and obesity (19). One of the modifiable contributors to the onset of inflammation and related diseases is dietary consumption (20). Hence, considerable emphasis has been placed on investigating the inflammatory effects of various nutrients and food items as well as their potential to combat inflammation. Previous studies have proposed that consuming a diet resembling the Western-style, marked by intensified amounts of fat, salt, and refined carbohydrates, heightens the vulnerability to inflammatory conditions such as MetS (21, 22). Proinflammatory diets have been verified to exhibit significant connections with multiple inflammatory biomarkers, such as the counts of white blood cells, c-reactive protein, interleukin-6, and tumor necrosis factor-alpha (23). Conversely, a dietary pattern marked by an elevated intake of whole grains and fruits (including whole grains, fruits, nuts, and dark leafy vegetables) was discovered to have an inverse relationship with CRP, IL-6, and homocysteine levels (24, 25). Meanwhile, the consumption of diets containing a high DII score can disrupt the balance of gut microbiota, resulting in the growth of detrimental bacteria and the production of an excessive amount of endotoxins. Subsequently, these toxins infiltrate the circulatory system, eliciting mild inflammation and inducing metabolic disruption within the body (26).

In our study, we discovered that diets that have a high proinflammatory effect are generally not considered healthy. The individuals who followed such diets tended to consume higher amounts of saturated fatty acids. These fatty acids have been found to trigger an inflammatory response in the body by increasing the reservois of TG in adipose tissue (27). Additionally, these individuals consumed fewer antioxidants, which play a crucial role in maintaining the redox balance in the body (28). As a result, the lack of antioxidants led to damage to the endothelial cells, thereby further exacerbating the inflammatory response. Notably, the consumption of red meat was also commonly observed among these individuals, and this dietary habit has been associated with the heightened presence of soluble adhesion molecules (25). Inflammation has been associated with the consumption of soft drinks that are high in fructose content. Fructose initiates oxidative stress and activates NF-κB, resulting in the induction of a stress response in the liver and causing dysregulation in lipid metabolism (29, 30). Adipose tissue has been established as a crucial contributor to the synthesis of inflammatory cytokines, a phenomenon that potentially underlies the high incidence of insulin resistance and diabetes among individuals with obesity (31–33). Our investigation revealed that individuals adhering to a diet that promotes inflammation experienced elevated BMI and increased rates of obesity. These findings suggest that such dietary patterns are more inclined to facilitate weight gain. Consequently, this could potentially disrupt the delicate equilibrium between the immune system and metabolism, resulting in the onset of MetS.

In recent years, there has been increasing research indicating that a healthy diet plays a crucial role in non-pharmacological interventions for patients MetS (34). According to our results, clinicians may utilize the DII in a clinical setting to assess the dietary habits of individuals with MetS and should encourage these patients to adopt an anti-inflammatory diet. Various dietary approaches, such as the Dietary Approaches to Stop Hypertension and Mediterranean diets—which emphasize whole grains, low-fat dairy products, legumes, nuts, and olive oil—have been shown to be beneficial for individuals with MetS (35, 36). Although our study cannot definitively determine which specific diet is the most advantageous for those with MetS, the aforementioned dietary patterns have been associated with anti-inflammatory effects, thereby providing further support for the recommendation of a low-DII diet for this population.

Our study is the first to comprehensively investigate the relationship between the DII and mortality among adults with MetS. Our research utilized a substantial and nationally representative sample of the American population. Moreover, detailed analyses were carried out, taking into account various covariates including demographics, examination-related factors, and laboratory measurements. The reliability of our results was confirmed by conducting sensitivity and subgroup analyses, thereby reinforcing our findings. This study aimed to provide novel insights into significant aspects of public health, including dietary management, nutritional epidemiology, and treatment for MetS.

It is imperative to recognize the constraints of our study. Firstly, while we took measures to minimize reverse causation by excluding individuals who passed away within a 2-year follow-up period, the observational nature of our study prevents us from establishing a conclusive causal link between DII and all-cause mortality. Secondly, the DII we computed based on dietary recall interviews conducted within 24 hours only offers a partial depiction of habitual eating patterns. It is essential to recognize that the data collected through questionnaires, encompassing dietary interviews, self-reported diagnoses, and smoking habits, could introduce recall biases. Third, although most relevant confounders have been addressed, some residual confounders may still persist. Fourth, this study evaluated the initial DII score and its correlation with prognosis; however, it is crucial to conduct dynamic monitoring of DII scores throughout the follow-up period. Ultimately, even though NHANES experienced a substantial rate of involvement, it still encountered situations where certain variables had missing values. This circumstance possesses the potential to jeopardize the representative nature of our sample.

## Conclusions

According to our research, a correlation has been discovered between DII and mortality in individuals diagnosed with MetS. This implies that consumption of a proinflammatory diet might elevate the likelihood of mortality in this specific patient population. The potential usefulness of utilizing the DII in evaluating dietary patterns for future public health management is emphasized. However, it is vital to carry out additional extensive prospective studies to establish a conclusive connection between an inflammatory diet and the initiation of MetS. In general, there is an urgent requirement to devise efficient approaches to prevent and manage chronic diseases on a broader scope.

## Data Availability

Publicly available datasets were analyzed in this study. This data can be found here: https://www.cdc.gov/nchs/nhanes/index.htm.
