# Supplementary material for: The relationship between dietary inflammatory index and all-cause and cardiovascular disease-related mortality in adults with metabolic syndrome: a cohort study of NHANES
Source: Front Endocrinol (Lausanne). 2025 Jan 10;15:1417840. doi: 10.3389/fendo.2024.1417840 (PMC11757130; doi:10.3389/fendo.2024.1417840)
Supplement: Supplementary file 1 [file DataSheet1.docx]

**Supplementary method: The method of the calculation of Dietary Inflammatory Index**

Using data from the 24-hour period preceding the interview, the skilled personnel managed to approximate the overall consumption of calories, nutrients, as well as non-nutrient constituents in foods and beverages. After a 24-hour period, the individuals were instructed to fulfill a brief survey aimed at ascertaining the regularity or peculiarity of their dietary intake the day prior, the origin of the tap water they consumed, their application of salt, utilization of specialized diets, and the frequency with which they consumed fish and shellfish in the last month. The impact of dietary intake on inflammation is assessed through the measurement of 27 different nutrients in the DII. This evaluation involves the aggregation of scores assigned to each component of the diet consumed within a 24-hour period, encompassing both pro-inflammatory and anti-inflammatory diets. A Z-score can be calculated by subtracting the global daily mean intake and dividing by the standard deviation, and then the result is converted to a percentile score by doubling each percentile score and subtracting “1” to produce a symmetrical distribution. By multiplying the percentile value by the corresponding “overall inflammation effect score”, we can produce an individual “overall DII score”.
