# Supplementary material for: The relationship between dietary inflammatory index and all-cause and cardiovascular disease-related mortality in adults with metabolic syndrome: a cohort study of NHANES
Source: Front Endocrinol (Lausanne). 2025 Jan 10;15:1417840. doi: 10.3389/fendo.2024.1417840 (PMC11757130; doi:10.3389/fendo.2024.1417840)
Supplement: Supplementary file 2 [file Table1.docx]

**Table S1 The detail definition and classification of covariates.**

| **Variables** | **Definitions or Classification** |
| --- | --- |
| **Sex** | Male, Female |
| **Race** | Non-Hispanic White, Others |
| **Educational attainment** | Under high school, College or higher, High school or equivalent |
| **Family PIR** | PIR<1, PIR 1~2.9, PIR≥3 |
| **BMI** | BMI < 25 kg/m2; 25 ≤ BMI ≤ 30 kg/m2; BMI > 30 kg/m2. |
| **Smoking status^1^** | Never smoking: <100 cigarettes in lifetime;  Former smoking: >100 cigarettes in life and smoke not at all now;  Now smoking: >100 cigarettes in lifetime. |
| **Alcohol drinking^2^** | Never : less than 12 drinks in lifetime.  Former: more than 12 drinks in lifetime but not consumed any alcohol in the past year.  Mild: women who consumed one drinks per day or men who consumed two drinks per day.  Moderate: women who consumed two or more drinks per day or men who consumed three or more drinks per day.  Heavy: as women who consumed three or more drinks per day or men who consumed four or more drinks per day, with at least five or more binge drinking episodes per month. |
| **IFG** | 6.1 mmol/l ≤ Fasting glucose ≤ 7.0 mmol/l. |
| **IGT** | 7.8 mmol/l ≤ Two-hour OGTT blood glucose ≤ 11.1 mmol/l. |
| **Diabetes^3^** | (1) The participant responded affirmatively to the query 'Do you possess a definite diagnosis of DM? ';  (2) HbA1c levels surpassed 6.5%;  (3) fasting plasma glucose levels were equal to or greater than 7.0 mmol/L;  (4) randomly assigned blood glucose levels were equal to or greater than 11.1 mmol/L;  (5) levels from the 2-hour oral glucose tolerance test were equal to or greater than 11.1 mmol/L;  (6) the participant was undergoing diabetes medication or insulin therapy. |
| **Hypertension^4^** | Hypertension was defined as a systolic blood pressure ≥ 140 mmHg and/or diastolic blood pressure ≥ 90 mmHg, or as having received antihypertensive treatment. |
| **CVD** | self-reported heart disease, congestive heart failure, heart attack, stroke or angina |
| **Hyperlipidemia** | Hypertriglyceridemia: TG ≥ 150mg/dl;  Hypercholesterolemia: TC ≥ 200mg/dl, LDL ≥ 130mg/dl;  Low HDL-C: HDL-C < 40mg/dl (male),50mg/dl (female);  Use of lipid-lowering drugs. |
| **Laboratory tests** | The Specific method can be found in this webpage  (https://wwwn.cdc.gov/nchs/nhanes/continuousnhanes/labmethods.aspx?BeginYear=2017) |

Abbreviation: PIR, family poverty income ratio; BMI, Body Mass Index; IFG, impaired fasting glycaemia; IGT, impaired glucose tolerance; OGTT, oral glucose tolerance test; HDL-C, high-density lipoprotein cholesterol; CVD, Cardiovascular Diseases; TG, Triglycerides; TC, total cholesterol; DM, Diabetes; HbA1c, Glycated Hemoglobin.

**References**

1. Beddhu S, Baird BC, Zitterkoph J, Neilson J, Greene T. Physical activity and mortality in chronic kidney disease (NHANES III). *Clin J Am Soc Nephrol*. Dec 2009;4(12):1901-6. doi:10.2215/CJN.01970309

2. Li XY, Wen MZ, Xu YH, Shen YC, Yang XT. The association of healthy eating index with periodontitis in NHANES 2013-2014. *Front Nutr*. 2022;9:968073. doi:10.3389/fnut.2022.968073

3. Wan Z, Guo J, Pan A, Chen C, Liu L, Liu G. Association of Serum 25-Hydroxyvitamin D Concentrations With All-Cause and Cause-Specific Mortality Among Individuals With Diabetes. *Diabetes Care*. Feb 2021;44(2):350-357. doi:10.2337/dc20-1485

4. Xiong YJ, Du LL, Diao YL, et al. Association of dietary inflammatory index with helicobacter pylori infection and mortality among US population. *J Transl Med*. Aug 12 2023;21(1):538. doi:10.1186/s12967-023-04398-8
