# Supplementary material for: The relationship between dietary inflammatory index and all-cause and cardiovascular disease-related mortality in adults with metabolic syndrome: a cohort study of NHANES
Source: Front Endocrinol (Lausanne). 2025 Jan 10;15:1417840. doi: 10.3389/fendo.2024.1417840 (PMC11757130; doi:10.3389/fendo.2024.1417840)
Supplement: Supplementary file 3 [file Table2.docx]

**Table S2 Baseline characteristics according to all-cause mortality^1^.**

| **Characteristic** | **Overall(N=13,751)** | **Alive(N=11,408)** | **Death(N=2,343)** | ***P* value** |
| --- | --- | --- | --- | --- |
| **Age，years** | 53.74(0.22) | 51.62(0.23) | 67.41(0.45) | < 0.0001 |
| **Sex, n (%)** |  |  |  | 0.06 |
| Female | 7449(52.95) | 6323(53.34) | 1126(50.44) |  |
| Male | 6302(47.05) | 5085(46.66) | 1217(49.56) |  |
| **Educational attainment** |  |  |  | < 0.0001 |
| Under high school | 4086(19.20) | 3159(17.30) | 927(31.42) |  |
| High school or equivalent | 7399(59.36) | 6255(60.04) | 1144(54.98) |  |
| College or higher | 2266(21.44) | 1994(22.65) | 272(13.60) |  |
| **Race, n (%)** |  |  |  | < 0.0001 |
| Non-Hispanic White | 6435(70.72) | 4948(69.19) | 1487(80.56) |  |
| Others | 7316(29.28) | 6460(30.81) | 856(19.44) |  |
| **Family PIR** |  |  |  | < 0.0001 |
| PIR<1 | 2901(14.37) | 2423(14.18) | 478(15.58) |  |
| PIR 1~2.9 | 6242(39.72) | 4976(37.91) | 1266(51.32) |  |
| PIR≥3 | 4608(45.92) | 4009(47.91) | 599(33.09) |  |
| **BMI, kg/m^2^** |  |  |  | < 0.0001 |
| BMI<25 | 896( 5.89) | 630( 5.04) | 266(11.38) |  |
| BMI 25~30 | 4124(28.53) | 3270(27.66) | 854(34.12) |  |
| BMI≥30 | 8731(65.58) | 7508(67.30) | 1223(54.50) |  |
| **Smoking status, n (%)** |  |  |  | < 0.0001 |
| Never | 6990(50.00) | 6060(51.90) | 930(37.81) |  |
| Former | 4109(30.34) | 3126(28.61) | 983(41.46) |  |
| Now | 2652(19.66) | 2222(19.49) | 430(20.73) |  |
| **Alcohol drinking** |  |  |  | < 0.0001 |
| Never | 2314(13.43) | 1870(12.85) | 444(17.15) |  |
| Former | 3116(19.87) | 2251(17.48) | 865(35.26) |  |
| Mild | 4340(35.16) | 3656(35.77) | 684(31.18) |  |
| Moderate | 1707(13.87) | 1550(14.88) | 157( 7.37) |  |
| Heavy | 2274(17.68) | 2081(19.02) | 193( 9.04) |  |
| **Diabetes diagnosis, n (%)** |  |  |  | < 0.0001 |
| DM | 5453(32.57) | 4183(29.88) | 1270(49.88) |  |
| IFG | 1123( 8.59) | 941(8.78) | 182(7.38) |  |
| IGT | 492( 3.63) | 425(3.66) | 67(3.43) |  |
| No | 6683(55.21) | 5859(57.68) | 824(39.30) |  |
| **Hypertension diagnosis, n (%)** | 9278(64.55) | 7351(61.96) | 1927(81.21) | < 0.0001 |
| **CVD diagnosis, n (%)** | 2607(16.20) | 1661(12.62) | 946(39.31) | < 0.0001 |
| **Hyperlipidemia diagnosis,n(%)** | 12907(94.52) | 10721(94.54) | 2186(94.37) | 0.79 |
| **Dietary inflammatory index** | 1.62(0.03) | 1.59(0.03) | 1.82(0.05) | < 0.0001 |
| **Waist circumference (cm)** | 110.69(0.20) | 110.76(0.22) | 110.21(0.45) | 0.26 |
| **GGT(U/L)** | 34.68(0.49) | 34.02(0.49) | 38.90(1.81) | 0.01 |
| **SBP (mmHg)** | 128.32(0.24) | 127.43(0.27) | 134.05(0.65) | < 0.0001 |
| **DBP (mmHg)** | 73.11(0.22) | 73.97(0.22) | 67.54(0.49) | < 0.0001 |
| **HbA1c(%)** | 6.05(0.02) | 6.00(0.02) | 6.34(0.04) | < 0.0001 |
| **Albumin(g/dl)** | 4.20(0.01) | 4.21(0.01) | 4.12(0.01) | < 0.0001 |
| **ALT(U/L)** | 28.66(0.31) | 29.01(0.25) | 26.37(1.55) | 0.09 |
| **AST(U/L)** | 26.11(0.18) | 26.02(0.19) | 26.72(0.52) | 0.21 |
| **Serum creatinine(umol/l)** | 81.40(0.38) | 78.74(0.38) | 98.53(1.30) | < 0.0001 |
| **Uric acid (umol/l)** | 351.18(1.10) | 348.07(1.20) | 371.19(2.38) | < 0.0001 |
| **HDL-C(mmol/l)** | 1.13(0.00) | 1.13(0.00) | 1.18(0.01) | < 0.0001 |

^1^Continuous variables are expressed as the mean (standard error). Categorical variables are expressed as unweighted numbers (weighted percentages). All estimates take into account the complex survey design.

BMI, body mass index; SBP, systolic blood pressure; DBP, diastolic blood pressure; PIR, family poverty income ratio; IFG, impaired fasting glycaemia; IGT, impaired glucose tolerance; DM, diabetes; HbA1c glycated hemoglobin; ALT, alanine aminotransferase; AST, aspartate aminotransferase; CVD, cardiovascular diseases; GGT, gamma-glutamyl transpeptidase; HDL-C, high-density lipoprotein cholesterol
